# Pigment epithelium-derived factor is an interleukin-6 antagonist in the RPE: Insight of structure-function relationships

**DOI:** 10.3389/fphys.2022.1045613

**Published:** 2022-11-16

**Authors:** Alexandra Bernardo-Colón, Miriam Lerner, S. Patricia Becerra

**Affiliations:** Laboratory of Retinal Cell and Molecular Biology, Section of Protein Structure and Function, National Eye Institute, National Institutes of Health, Bethesda, MD, United States

**Keywords:** interleukin-6 (IL-6), pigment epithelium-derived factor (PEDF), tumor necrosis factor, alpha, retinal pigment epithelium (ARPE-19), inflammation

## Abstract

Retinal and choroidal inflammatory lesions increase the levels of the pro-inflammatory cytokine interleukin-6 (IL-6). Pigment epithelium-derived factor (PEDF) has anti-inflammatory properties, but it is not known if it can prevent the production of IL-6 by the retinal pigment epithelium. To investigate the anti-inflammatory effects of PEDF in the RPE, we used human ARPE-19 cells stimulated with human recombinant tumor necrosis factor-alpha (TNF-α) to induce overexpression of the *IL6* gene. We found that the viability of ARPE-19 cells decreased by 22% with TNF-α at 10 ng/ml, being drastically decreased at ≥50 ng/ml. TNF-α at 5–100 ng/ml elevated the production and secretion of IL-6 protein, as measured by ELISA. To challenge the TNF-α-mediated stimulation of IL-6, we used recombinant human PEDF protein. PEDF at 100 nM recovered the TNF-α-mediated loss of cell viability and repressed *IL-6* gene expression as determined by RT-PCR. PEDF at 10–100 nM attenuated the IL-6 protein secretion in a dose dependent fashion (IC50 = 65 nM), being abolished with 100 nM PEDF. To map the region that confers the IL-6 blocking effect to the PEDF polypeptide, we used chemically synthesized peptides designed from its biologically active domains, pro-death 34-mer, and pro-survival 44-mer and 17-mer (H105A), to challenge the IL-6 overproduction. The pro-survival peptides recovered the TNF-α-mediated cell viability loss, and inhibited IL-6 secretion, while the 34-mer did not have an effect, suggesting a role for the pro-survival domain in blocking TNF-α-mediated cell death and IL-6 stimulation. Our findings position PEDF as a novel antagonistic agent of IL-6 production in RPE cells, underscoring its use for the management of retinal disease-related inflammation.

## Introduction

Retinal and choroidal inflammatory lesions due to injury, infection, or disease, can eventually cause visual loss. Inflammatory cytokine infiltration of the choroid, retina and vitreous is a pathogenic feature of a spectrum of inflammatory, degenerative, and dystrophic diseases of the retina and choroid. In retinal degeneration diseases, a group of heterogeneous disorders, which include age-related macular degeneration (AMD) and retinitis pigmentosa (RP), neuroretinal inflammation participates as a stimulant promoting cell damage leading to progressive photoreceptor cell loss and ultimately to blindness (Wert et al., 2014). The retinal pigment epithelium (RPE) consists of a monolayer of cells located between the choroid and the neural retina. As part of the blood-retinal barrier, the RPE is positioned to interact with neural cells in the retina and inflammatory cytokines emerging from choroidal vessels, thereby enabling it to perform critical roles in the initiation and propagation of ocular inflammation. RPE cells are essential for preserving retina homeostasis, and when dysfunctional they can contribute to the development of retinal dystrophies through their exacerbated inflammatory response. Therefore, there is great interest to understand the mechanisms related to controlling the choroiretinal inflammatory processes by the RPE (Ambati et al., 2003; Fritsche et al., 2014; Kaur and Singh, 2021).

Interleukin-6 (IL-6) is a cytokine secreted by RPE cells upon inflammatory stimulation that leads to cellular and tissue damage in a variety of pathologies of the retina (Takeda et al., 2020). IL-6 plays a role in immune response and inflammation, as well as in wound healing response and angiogenesis (Kishimoto, 2010). RPE cells in culture secrete IL-6 when stimulated with other cytokines, such as tumor necrosis factor-α (TNF-α), interleukin-1-β (IL-1-β), etc., gradually accumulating IL-6 in media over subsequent time in culture (Elner et al., 1992; Planck et al., 1992; Holtkamp et al., 1998).

The RPE also releases pigment epithelium-derived factor (PEDF) into the interphotoreceptor matrix where it acts on retinal neuroprotection (Becerra et al., 2004; Polato and Becerra, 2016). PEDF is a glycoprotein belonging to the superfamily of serine protease inhibitors (SERPIN) (Becerra, 2006). It has demonstrable anti-inflammatory properties: PEDF suppresses the expression of pro-inflammatory cytokines in endothelial cells, in the pathogenesis of dry eye disease, and in lysophosphatidic acid (LPA)-stimulated granulosa cells of the ovary (hyperandrogenic Polycystic ovary syndrome) (Yamagishi et al., 2004; Miller et al., 2016; Miller et al., 2020; Ma et al., 2021). PEDF overexpression attenuates the expression of hepatic *Il6* gene induced in dietary steatohepatitis of mice (Yoshida et al., 2017), in atherosclerotic plaque of *ApoE* ^*−/−*^ mice *in vivo,* and in LPS-stimulated RAW264.7 cells *in vitro* (Wen et al., 2017). In contrast, recombinant human PEDF truncated from the NH_2_-end upregulated inflammatory-related *Il6* gene in cultured microglial cells from rat brain during the 3–9 h after addition but declined by 24 h (Sanagi et al., 2005), and in cultured rat neonatal astrocytes (Yabe et al., 2005). PEDF has two reported biologically active domains, pro-survival and pro-death domains, which promote neurotrophic activity and prevent angiogenesis, respectively (Becerra, 2006) (Amaral and Becerra, 2010) (Kenealey et al., 2015). However, the effects of PEDF and these domains in TNF-α-stimulated RPE cells have not been reported yet.

In this study, we hypothesize that PEDF can repress *IL6* gene expression and decrease the IL-6 protein release by RPE cells. We used the human ARPE-19 cell line and stimulated it with TNF-α to test the effects of human recombinant PEDF protein and synthetic PEDF peptides derived from its biologically active domains on secretion of IL-6 to the culture media. We found that PEDF effectively blocked IL-6 production. We discuss PEDF as a novel agent for potential use in retinal disease-related inflammation management due both to its presence in the RPE, and its role in suppressing IL-6.

## Materials and methods

### Cell culture

Human ARPE-19 cells (ATCC, Manassas, VA, United States, Cat. # CRL-2302) were maintained in Dulbecco’s modified eagle medium/Nutrient Mixture F-12 (DMEM/F-12) (Gibco; Grand Island, NY, Cat. #11330-032) supplemented in 10% fetal bovine serum (FBS) (Gibco; Grand Island, NY Cat#10082-147) and 1% penicillin/streptomycin (Gibco; Grand Island, NY Cat.# 15070-063) at 37°C with 5% CO as previously described (Bullock et al., 2021). For assays described below, a total of 0.2 × 10^6^ cells in 0.5 ml were plated per well of a 24-well plate and incubated for 72 hours in DMEM/F12 and 1% penicillin-streptomycin and no FBS before cell treatment. ARPE-19 cells were authenticated by Bio-Synthesis, Inc. (Lewisville, TX) at passage 27. ARPE-19 cells in passage numbers 27–32 were used for all experiments.

### Cell viability

Nexcelom Auto T4 automated cell counter (Bioscience, Lawrence, MA) was used to determine viability of cells. Briefly, as described before (Bullock et al., 2021), cells were detached with 0.25% Trypsin-EDTA (Gibco, Cat# 25200-072) with 2 min incubation at 37°C, transferred to 1.5-ml Eppendorf tubes, washed twice with 0.5 ml PBS (Quality Biological, Cat# 119-069-101) and centrifuged at 1,040 × g for 4 min. The cells were stained with 0.1% trypan blue (Gibco, Cat# 152550-061) and cell viability was measured by dye exclusion method. Data was analyzed by the Cellometer software and percent viability was obtained.

Crystal violet staining was also used to determine cell viability, as described before (Subramanian and Becerra, 2019; Bullock et al., 2021). Media from the wells were removed, and the cells were washed twice with distilled water. The plate was inverted to remove excess water. After at least 2 h at room temperature, 50 µL of 0.1% crystal violet staining solution (in 25% methanol, filtered before use) were added to each well and incubated for 30 min at 25 °C with shaking at 20 oscillations per min. For crystal violet extraction, 200 uL of methanol was added to each well, and the plate was covered with a lid and incubated at room temperature for 20 min on a rocker set at 20 oscillations per minute. Absorbance of the extracted crystal violet was measured at 570 nm using an EnVision Plate Reader (Perkin Elmer, Waltham, MA).

### Treatment with TNF-α

ARPE-19 cells were seeded as described before (Gambhir et al., 2012) at 0.2 × 10^6^ cells per well of 24 well-plates in culture media without FBS. Seventy-2 hours (72) after seeding, cells were treated with either TNF-α (Sigma Aldrich, Cat# T6674), as previously described (Gambhir et al., 2012) or a combination of TNF-α plus PEDF or 34-mer, 44-mer and 17-mer (H105A) peptides for 24 h. Concentrations of the factors in each experiment are indicated in the legends. Following treatment, the cell media were collected for ELISA and cells were harvested for total RNA isolation for use in RT-PCR and qPCR experiments.

### RNA extraction, cDNA synthesis, and quantitative RT-PCR

Total RNA was purified using RNeasy^®^ Mini Kit for ARPE-19 cells (Qiagen, Cat. # 74104) as previously described (Bullock et al., 2021). Briefly, a total of 10 ng of RNA were used for reverse transcription using the SuperScript III first-strand synthesis system (Invitrogen, Cat. # 18080-051). The human *IL6* transcript levels in ARPE-19 cells determined by quantitative RT-PCR were normalized using the QuantiTect SYBR Green PCR Kit (Qiagen, Cat. # 204143) in the QuantStudio 7 Flex Real-Time PCR System (Thermo Fisher Scientific, Waltham, MA). The primer sequences included in this study are found listed in Table 1. *IL6* relative expression to *18S* was calculated using the comparative ΔΔC method (Gambhir et al., 2012).

**TABLE 1:** Primer sequences.

*Gene name* (protein)	Accession #	Forward primer	Reverse primer
*Rps18* (ribosomal protein S18)	NM_022551	5′-GGT​TGA​TCC​TGC​CAG​TAG-3′	5′-GCG​ACC​AAA​GGA​ACC​ATA​AC-3′
Human IL-6	NC_000007.14	5′-AAC​CTG​AAC​CTT​CCA​AAG​ATG​G-3′	5′-TCT​GGC​TTG​TTC​CTC​ACT​ACT-3′

### ELISA

A Quantikine Human Interleukin (IL-6) ELISA was used according to the manufacturer’s directions (R&D Systems Inc., Minneapolis, MN, United States, Cat. #D6050) to determine the IL-6 concentration in the cell culture media. Inter and intra-variability were previously demonstrated to be low for these kits (Gambhir et al., 2012). IL-6 concentrations were calculated from the comparison of absorbance of the samples and the ELISA IL-6 standard curve obtained with pure IL-6 at 0–300 pg/ml.

## Results

### PEDF promoted ARPE-19 cell viability against TNF-α

**FIGURE 1:**
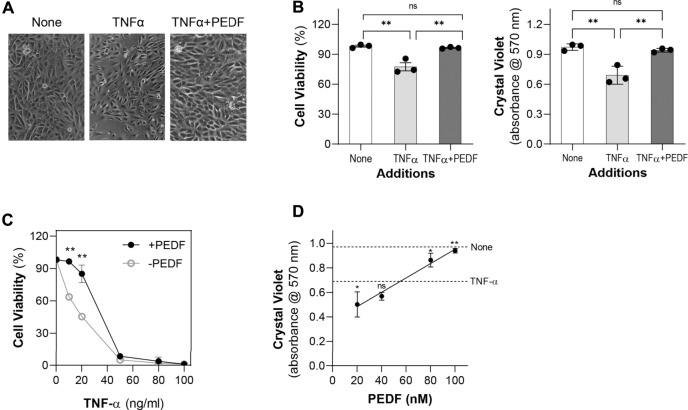
PEDF promoted ARPE-19 cell viability against TNF-α **(A)** Representative images of ARPE-19 cells 24 h post treatment with no factor (None), TNF-α (10 ng/ml), and TNF-α (10 ng/ml) + PEDF (100 nM). **(B)** Quantification of cell viability from triplicate wells per condition and repeated in three experiments using the automated cell viability method (left) and the Crystal Violet staining method (right). **(C)** Quantification of cell viability of ARPE-19 cells treated with increasing concentrations of TNF-α (as indicated in the *x*-axis) without (○) and with (●) 100 nM PEDF (as indicated in the plot) for 24 h. **(D)** Quantification of cell viability of ARPE-19 cells treated with increasing concentrations of TNF-α (as indicated in the *x*-axis) without (○) and with (●) 100 nM PEDF (as indicated in the plot) for 24 h using crystal violet staining. Each data point corresponds to the average of 3 wells per condition, for a total of 3 experiments. **p < 0.001.

To investigate the effects of PEDF on IL-6 overexpression in RPE cells, we treated human ARPE-19 cells in serum-free media with recombinant human TNF-α for 24 h. We noticed that with 10 ng/ml of TNF-α the viability of the cells decreased. However, a combination of 10 ng/ml of TNF-α and 100 nM recombinant human PEDF recover the cell viability to control levels without any factors. Figure 1A shows representative images of cells treated with no factors (None), TNF-α and TNF-α + PEDF. With both TNF-α and PEDF, the cells appeared healthier, and more viable than cells treated with only TNF-α (See Figure 1A, right image). Figure 1B shows quantification of cell viability (automated and crystal violet staining methods) from triplicate wells from each of the three conditions, which demonstrates that TNF-α at 10 ng/ml decreased the viability levels of RPE cells by 22% and 28% automated and by crystal violet assays, respectively, while 100 nM PEDF combined with TNF-α increased them to be similar to the controls without TNF-α (for automated, None, 98.13 ± 0.97%; TNF-α, 77.4 ± 4.10%; TNF-α *+*PEDF, 96.42 ± 0.51%; for crystal violet, None, 0.91 ± 0.01 absorbance units (AU); TNF-α, 0.68 ± 0.09 AU; TNF-α *+* PEDF 0.93 ± 0.01 AU; Ordinary one way-ANOVA *p*-value < 0.0001).

We then examined cells treated with increasing concentrations of TNF-α ranging from 5 ng/ml to 100 ng/ml. Examination of the cells under the microscope revealed that viability was compromised as the concentration of the TNF-α increased (not shown). TNF-α concentrations of 5–20 ng/ml allowed cells to remain adhered to the plate, although the cells appeared unhealthy, and viability decreased. At ≥50 ng/ml TNF-α, the viability of ARPE-19 cells drastically decreased (Figure 1C). However, additions of 100 nM PEDF prevented the decrease in cell viability caused by 5–20 ng/ml, but not with TNF-α at ≥50 ng/ml. PEDF dose response of ARPE-19 cell viability in the presence of 10 ng/ml TNF-α measured was performed using the crystal violet staining assay and showed that PEDF between 20-100 nM increased the cell viability in a linear fashion (Figure 1D). However, with 20 nM PEDF the cell viability was below, and with 80–100 nM PEDF above the cell viability of 10 ng/ml TNF-α. These observations indicated that PEDF at over 80 nM protected ARPE-19 cells against insult of the TNF-α up to 50 ng/ml.

### PEDF blocked TNF-α induced IL-6 overproduction of ARPE-19 cells

**FIGURE 2:**
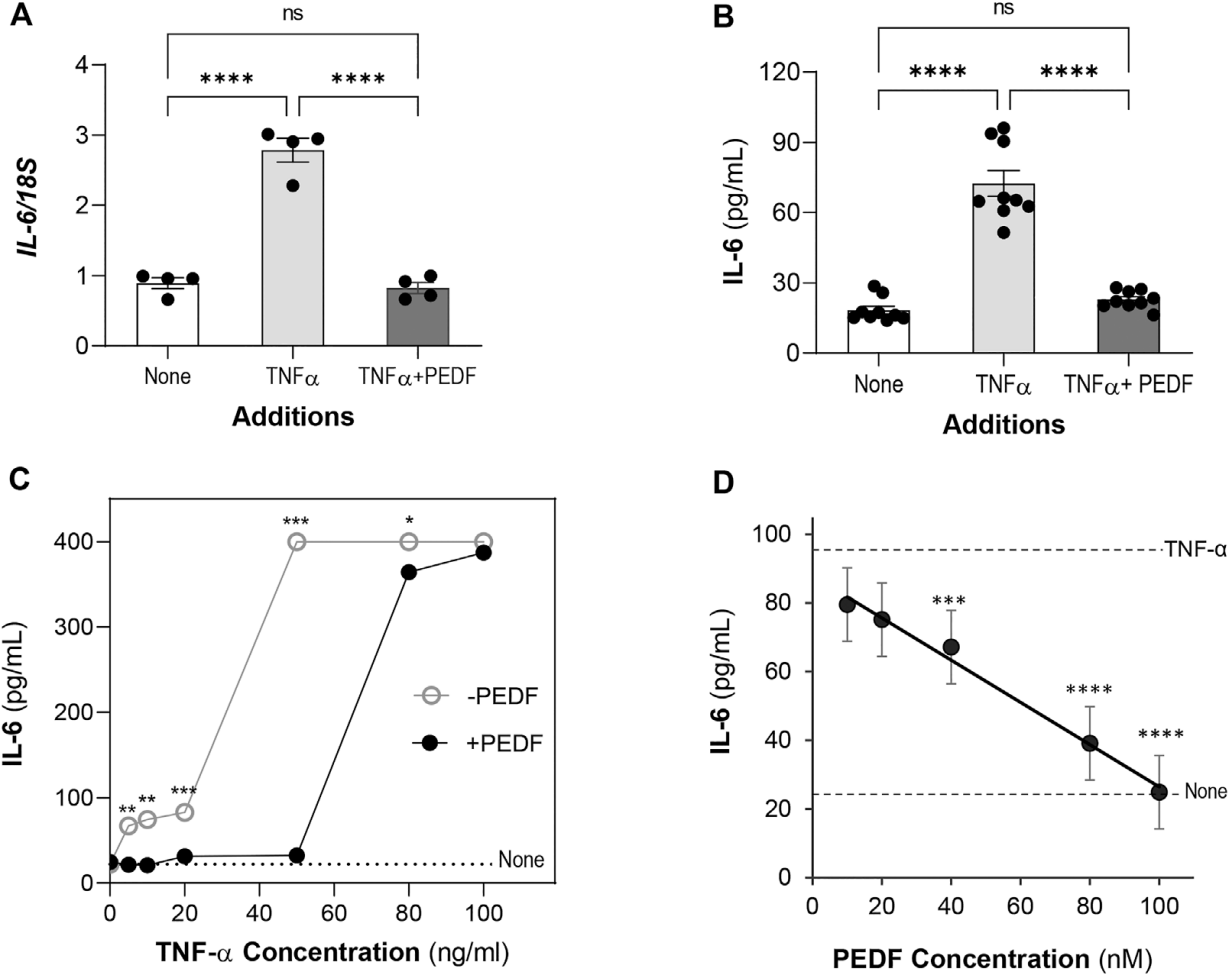
PEDF decreases IL-6 levels. ARPE-19 cells were treated with TNF-α or TNF-α + PEDF for 24 h. **(A)** Relative *IL6* gene expression in cells treated with no factor (None), 10 ng/ml TNF-α and 100 nM PEDF (as indicated in the *x*-axis) for 24 h was determined by RT-PCR using *18S* as a housekeeping gene control. Expression levels were normalized to *18S* and given as *IL6/18S* (*y*-axis). Each bar corresponds to an average of assays in quadruplicate wells. All the conditions had a distinct distribution of the *IL-6/18S* ratios. **(B)** Concentrations of IL-6 protein in the culturing media of ARPE-19 treated as in Panel A were determined by ELISA. Each bar corresponds to the average of 3 wells per treatment and repeated in three experiments each with an independent batch of cells. **(C)** IL-6 concentration in media of cells treated with increasing concentrations of TNF-α (indicated in *x*-axis) without (○) and with (●) 100 nM PEDF (as indicated in the plot). The dotted line corresponds to the IL-6 concentration without any factor (None). Each data point corresponds to the average of IL-6 from 3 wells per treatment. **(D)** IL-6 concentration in media of cells treated with 10 ng/ml TNF-α and increasing concentrations of PEDF (indicated in *x*-axis). The linear trendline was determine using Excel. The dotted lines correspond to the IL-6 concentrations with 10 ng/ml TNF-α alone and no factors, None (as indicated to the right). Each data point corresponds to the average of IL-6 from 3 wells per treatment. *p < 0.05, **p < 0.001, ***p < 0.0001, ****p < 0.00001.

Then we determined the *IL6* gene expression in the treated cells. The relative mRNA levels of *IL*6 gene in ARPE-19 cells with 10 ng/ml TNF-α (2.78 ± 0.17) was about 3.1-fold the levels of the control cells without this factor (None, 0.89 ± 0.07) (Figure 2A). Additions of 100 nM PEDF repressed the *IL*6 expression (0.82 ± 0.07) induced by TNF-α to levels similar to controls.

We proceeded to determine the concentration of IL-6 protein secreted by the cells. Similarly, the concentration of IL-6 in media of cells treated with 10 ng/ml TNF-α (72.4 ± 5.47 pg/ml) was about 4-fold the concentration of the control cells (None, 18.3 ± 1.72 pg/ml), indicating an overproduction of about 54 pg/ml IL-6. However, addition of PEDF at 100 nM decreased the IL-6 to levels similar to those in the controls (22.8 ± 1.26 pg/ml) (Figure 2B). (Ordinary one way-ANOVA *p*-value < 0.0001). Together, the findings indicate that exogenous PEDF interfered at the transcriptional level to block the IL-6 protein production and secretion.

We determined the IL-6 protein levels secreted by ARPE-19 cells stimulated with TNF-α at 5–100 ng/ml for 24 h. While the IL-6 concentration remained 22.58 ± 2.61 pg/ml without TNF-α, with 5–20 ng/ml of TNF-α, IL-6 increased to 66–83 pg/ml, respectively, indicating over-production of the cytokine induced with increasing TNF-α concentrations. However, with TNF-α at ≥50 ng/ml the IL-6 concentrations plateaued at 400 pg/ml (Figure 2C), and the cells detached and lysed (not shown) precluding accurate determination of cytokine production. Because TNF-α at 10 ng/ml kept the cells alive and accumulated about 74 pg/ml IL-6 in the media by 24 h, we chose this concentration to induce IL-6 in ARPE-19 cells for the remainder of the experiments. Another set of cultures that received 100 nM PEDF in addition to 5–100 ng/ml of TNF-α showed that the IL-6 concentration decreased in each case, but PEDF did not lower the IL-6 concentration stimulated by TNF-α at 80 ng/ml and 100 ng/ml (400 pg/ml) (Figure 2C). The observations indicated that PEDF blocked IL-6 production in stimulated ARPE-19 cells with up to 50 ng/ml TNF-α.

### PEDF decreases IL-6 levels in a concentration-response fashion

We proceeded to assess a possible concentration-response relationship for the antagonistic effect of PEDF over the IL-6 overproduction by ARPE-19 cells. PEDF protein at concentrations ranging 10–100 nM and a constant concentration of TNF-α (10 ng/ml) were incubated in the ARPE-19 cell cultures for 24 h. Figure 2D shows that the IL-6 protein concentration in the culture media of TNF-α stimulated cells decreased with PEDF in a concentration-response fashion, with a calculated half-maximal inhibitory concentration (IC50) of 65 nM PEDF, and a maximum blocking concentration of 100 nM PEDF. PEDF at 100 nM abolished the IL-6 overproduction stimulated by 10 ng/ml TNF-α. The concentration-response relationship showed a negative correlation between PEDF and IL-6 overproduction by RPE cells stimulated by TNF-α.

### Effects of PEDF peptides on cell viability

**FIGURE 3:**
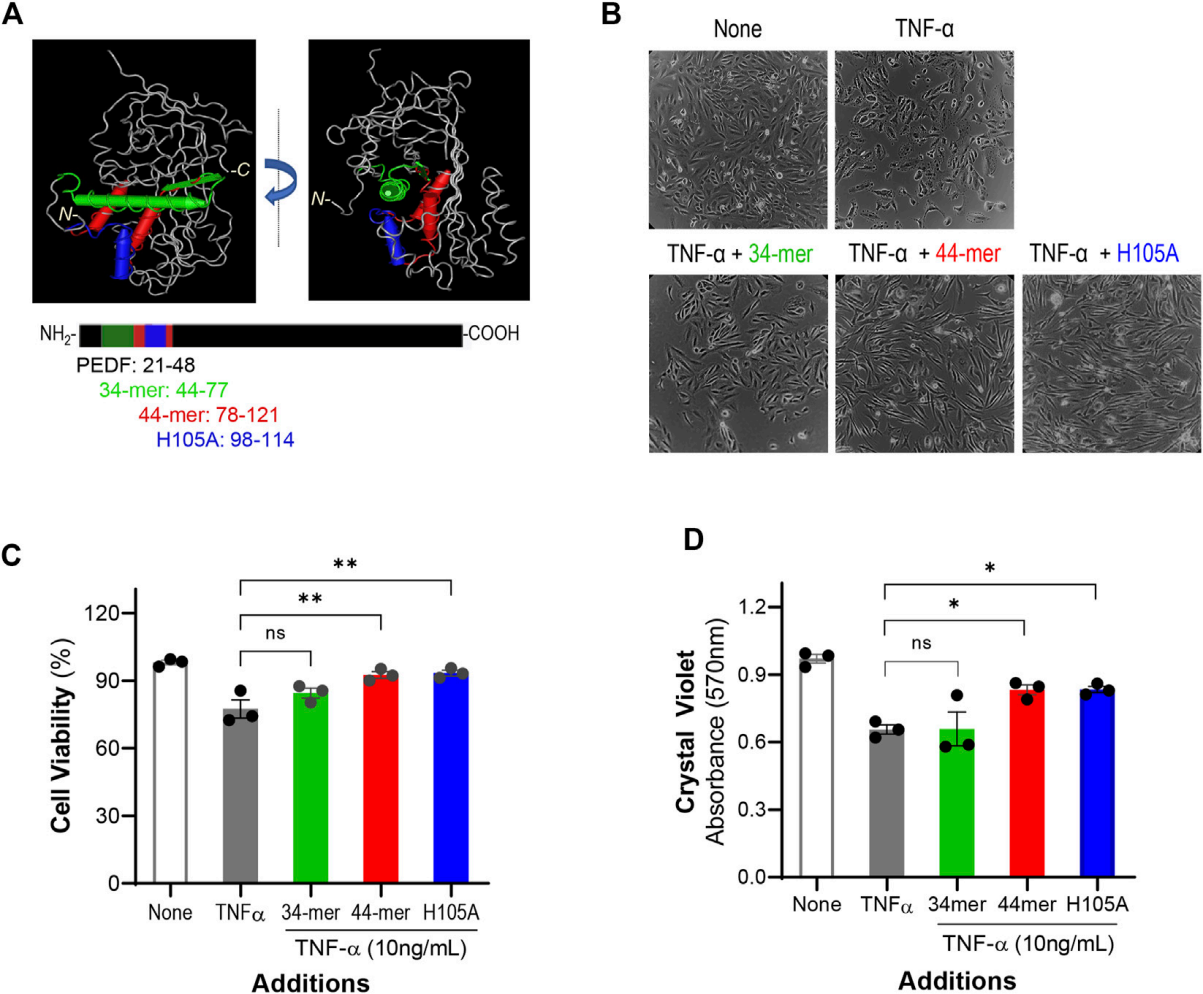
PEDF peptides effects on cell viability **(A)** 3D molecular model of human PEDF (PDB 1IMV) and the linear representation of PEDF polypeptide showing the location of the 34-mer (green), 44-mer (red), and 17-mer (H105A) (H105A, blue) domains. PEDF is given in grey in the upper images and in black in the lower illustrations. The amino acid positions for each peptide are given next to their names and color coded. **(B)** Representative images of ARPE-19 cells after 24 h of treatment with no factors (None), 10 ng/ml TNF-α, 10 ng/ml TNF-α + 10,000 nM 34-mer, 10 ng/ml TNF-α + 10,000 nM 44-mer, and 10 ng/ml TNF-α + 10,000 nM 17-mer (H105A), as indicated at the top of each image. **(C)** Quantification of the viability of cells as described in Panel **(B) (D)** Quantification of cell viability of ARPE-19 cells treated as described in Panel B using crystal violet staining. Each bar corresponds to the average of assays from 3 wells per treatment. ***p < 0.0001.

To identify the region of PEDF responsible for the IL-6 blocking effect, we used selected fragments of PEDF with known biological activities. Synthetic peptides designed from the pro-death 34-mer (positions 44–77), and the pro-survival 44-mer (78–121) and 17-mer (H105A) (98–114) domains of human PEDF were used to challenge the IL-6 overproduction. Figure 3A illustrates the location of the peptide regions in 3D spatial and linear models of human PEDF. The 34-mer and 44-mer are separate domains with distinct activities. The 17-mer (H105A) within the 44-mer region contains one alteration of the histidine in position 105 to alanine, which enhances the affinity for the receptor of PEDF and cytoprotective activity in retinal cells (Kenealey. J., 2015). ARPE-19 cells were treated with combinations of TNF-α and individual peptides for 24 h, and then the effects on the cells were assessed. Figures 3B,C show representative images of the treated cells and a plot with quantification of cell viability, respectively. As shown above, the cell viability without any factor (None, 98.1 ± 0.97%) was reduced with additions of TNF-α (77.4 ± 2.24%). However, the viability of cells treated with TNF-α + 44-mer (92.5 ± 1.50%) and TNF-α + H105A (93.3 ± 1.24%) was comparable to untreated cells (None), indicating that the pro-survival 44-mer and 17-mer (H105A) peptides protected against the TNF-α insult. The combination of TNF-α + 34-mer did not change the viability significantly (84.5 ± 2.17%) relative to the cultures with TNF-α alone. ARPE-19 cell viability measured using crystal violet staining with the peptides and 10 ng/ml TNF-α lead to similar conclusions (Figure 1D).

### Effects of PEDF peptides on IL-6 overproduction

**FIGURE 4:**
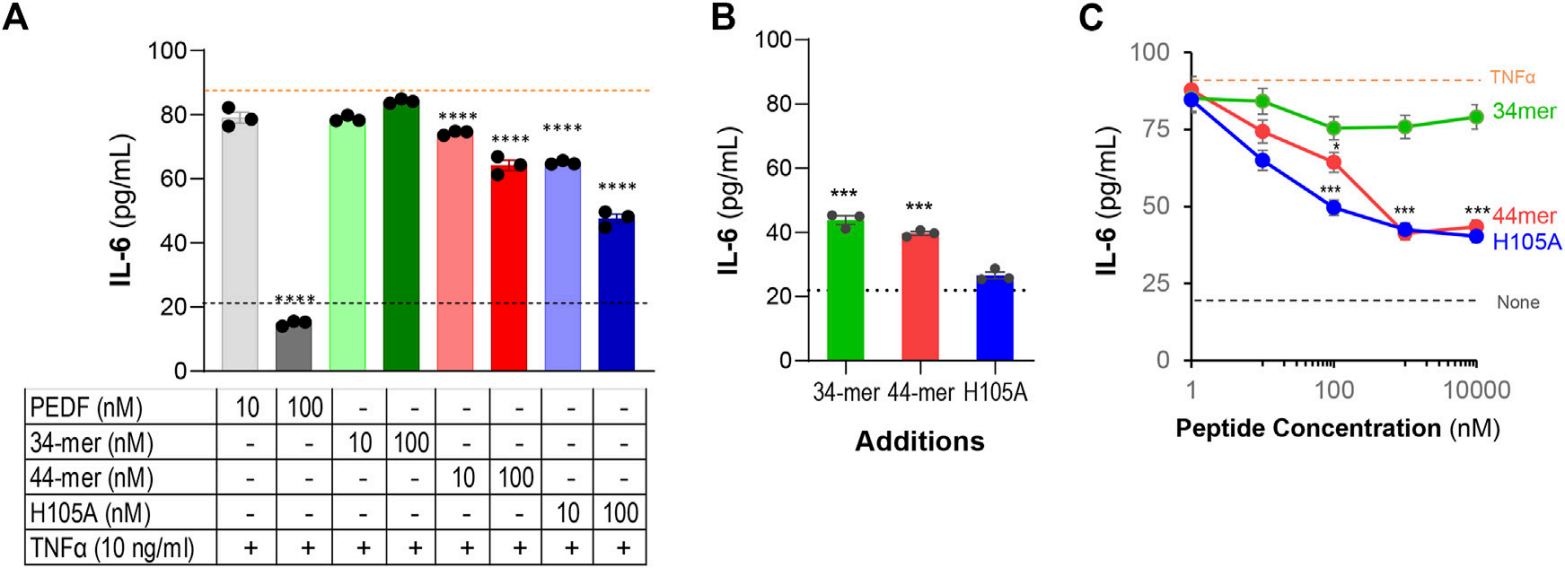
Effects of PEDF peptides on IL-6 overproduction. ARPE-19 cells were treated with TNF-α, PEDF peptides or TNF-α + PEDF peptides for 24 h and the IL-6 was determined in the culture media by ELISA. **(A)** Concentrations of IL-6 protein (*y*-axis) by ARPE-19 treated as indicated in the table below *x*-axis. Each bar corresponds to the average of assays from 3 wells per condition. The black and orange dotted lines correspond to the IL-6 concentration from media of cells without factors and with 10 ng/ml TNF-α alone, respectively. **(B)** Concentrations of IL-6 protein (*y*-axis) by ARPE-19 treated with 10,000 nM of only each peptide (no TNF-α) and as indicated in the *x*-axis. Each bar corresponds to the average of assays from 3 wells per condition. The black corresponds to the IL-6 concentration from media of cells without factors. **(C)** Concentrations of IL-6 protein (*y*-axis) by ARPE-19 treated with 10 ng/ml TNF-α in combination with increasing concentrations of peptides 34-mer (green), 44-mer (red), and 17-mer (H105A) (H105A, blue) at concentrations ranging 1 nM -10,000 nM, as indicated in the *x*-axis. Each data point corresponds to the average of 3 wells per condition. *p < 0.05, ***p < 0.0001, ****p < 0.00001.

We investigated the effects of the PEDF peptides on IL-6 stimulation by TNF-α in ARPE-19 cells. Figure 4A shows that additions of the 44-mer and 17-mer (H105A) peptides at 10 nM and 100 nM decreased the IL-6 concentration in the ARPE-19 media, while additions of the 34-mer peptide did not. To examine the possible stimulation of IL-6 by the peptides alone, we treated the cells with the peptides without TNF-α and determined the concentration of IL-6 in the culture media. Figure 4B shows that while the 34-mer and 44-mer peptides at 100-fold greater concentration (10,000 nM) increased marginally the IL-6 concentration (43.8 ± 1.35 pg/ml and 39.7 ± 0.58 pg/ml, respectively), the 17-mer (H105A) peptide did not (26.6 ± 1.07 pg/ml), suggesting the presence of an endogenous stimulant in samples of 34-mer and 44-mer.

We assessed a concentration-response relationship for the effects of each PEDF peptide over the IL-6 overproduction by ARPE-19 cells treated with TNF-α. ARPE-19 cells were incubated with peptides at concentrations ranging from 1-10,000 nM and a constant concentration of TNF-α (10 ng/ml) for 24 h. Figure 4C shows that IL-6 concentrations decreased with 44-mer and 17-mer (H105A) peptides in a concentration-response fashion, but not with the 34-mer peptide. The IC50 estimated from the concentration-response curves were 100 nM for the 44-mer and 10 nM for the 17-mer (H105A) peptide. The maximum blocking concentrations were obtained with ≥1,000 nM of each 44-mer and 17-mer (H105A), which lowered the IL-6 concentrations to 42.1 ± 1.93 pg/ml (average of the 4 points) from those obtained with TNF-α alone of 84.2 ± 0.38 pg/ml. We noted that the maximum blocking concentration of the full-length PEDF was 100 nM (see Figure 2D), which brought the IL-6 concentration to a lower level than the peptides. In contrast, peptide 34-mer at 10,000 nM lowered only to 79 pg/ml IL-6. We concluded that, similar to the full-length PEDF, the pro-survival 44-mer and 17-mer (H105A) blocked the stimulation of IL-6 in RPE cells, but at a lower efficacy, and the pro-death 34-mer did not have a significant effect.

## Discussion

In the present study, we demonstrated for the first time that PEDF protein blocked the cytokine IL-6 production by TNF-α stimulated RPE cells *in vitro*. The conclusion is based on the evidence that 1) human ARPE-19 cells overexpress the *IL6* gene and overproduce IL-6 protein and secrete it to the medium; and 2) human recombinant PEDF blocks the overexpression of *IL6* gene and overproduction of IL-6 protein by the TNF-α stimulated ARPE-19 cells. PEDF downregulates IL-6 at the transcriptional level. The fact that PEDF fragments 44-mer and 17-mer diminish the IL-6 secretion implies that the pro-survival domain confers the blocking effects to the PEDF polypeptide. Similar to the full-length PEDF, the survival activity of these peptides prevents the loss in cell viability of the TNF-α stimulated ARPE-19 cells. These observations highlight PEDF and its 44-mer and 17-mer peptides as potent suppressors of IL-6 cytokine production by the RPE and suggest their protective role against inflammatory-related retinal dystrophies.

The data presented here are dependent on the purity of the PEDF peptides. It is unlikely that the PEDF effects on ARPE-19 cells were non-specific because we have previously shown that the PEDF used was a highly purified and homogeneous protein (Bullock et al., 2022). In addition, the peptides used were synthesized chemically with a >95% purity and the HPLC analyses revealed only one peptide species in all cases. However, high concentrations (10,000 nM) of the synthetic peptides 34-mer and 44-mer by themselves induced certain overproduction of IL-6 protein, suggesting that they might either have a yet unknown contaminant to induce expression of the *IL-6* gene, which the 17-mer (H105A) does not have, or the 100-fold higher concentration of 34-mer and 44-mer peptides could induce off-target effects. While the estimated IC50 of 17-mer (H105A) (10 nM) is lower than that of PEDF (IC50 of 65 nM PEDF), 17-mer (H105A) needs to reach 1,000 nM or higher for maximum blocking effect, differing from PEDF, which abolishes the IL-6 overproduction at 100 nM. We compared the concentrations of PEDF used in our assays with those in a physiological environment. In this regard, the estimated human blood concentration of PEDF is 100–200 nM and the monkey and bovine interphotoreceptor matrix concentration of soluble PEDF is about 250 nM (Wu et al., 1995; Petersen et al., 2003; Yamagishi et al., 2006). These comparisons indicate that the concentrations of PEDF used in our experiments were within the physiological range. We note that peptide 17-mer (H105A) is not found naturally in the eye.

A possible molecular mechanism of antagonistic action of PEDF over IL-6 production by the RPE can be deduced from our findings. We envision that the 17-mer region of PEDF binds to the PEDF receptor PEDF-R on the surface of ARPE-19 cells (Kenealey et al., 2015) (Bullock et al., 2021). The binding stimulates the phospholipase A2 activity of PEDF-R to liberate preferentially docosahexaenoic acid as well as other omega-3 fatty acids (Notari et al., 2006; Subramanian et al., 2013), which become available to act as the mediators to repress *IL6* gene expression and decrease IL-6 production and secretion. Docosahexaenoic acid and other omega 3 fatty acids decrease the IL-6 overproduction (Song et al., 2017). Several lines of evidence point to the fact that long chain polyunsaturated fatty acids block the *IL6* gene expression (Honda et al., 2015) and decrease the production and secretion of IL-6 in cell cultures *in vitro*, in animals and in humans (Jia et al., 2004; SanGiovanni and Chew, 2005; Tikhonenko et al., 2013).

Our findings highlight the PEDF secreted by the RPE as a soluble factor with antagonistic effects over the cytokine IL-6. In this regard, PEDF can contribute to the immunomodulatory function of the RPE. In contrast, low retinal levels of PEDF, such as in retinal degenerations AMD and RP, and dystrophic RPE, would be permissive to inflammatory stimulation by increasing IL-6 and other proinflammatory factors infiltrating in the retina. In these cases, administration of exogenous PEDF or its 17-mer (H105A) peptide may serve to counteract such inflammatory stimulation of the retina to prevent progression of retinal degeneration. It will be of interest to explore if PEDF is also antagonistic to other proinflammatory cytokines of the retina. In summary, these findings position PEDF as a novel agent to downregulate IL-6 production in RPE cells, thereby underscoring its use for the management of retinal degeneration-related inflammation.

## Data Availability

The original contributions presented in the study are included in the article/supplementary material, further inquiries can be directed to the corresponding author.
